# Synergistic effects of natural products in combination with anticancer agents in prostate cancer: A scoping review

**DOI:** 10.3389/fphar.2022.963317

**Published:** 2022-09-12

**Authors:** Chunhoo Cheon, Seong-Gyu Ko

**Affiliations:** Department of Preventive Medicine, College of Korean Medicine, Kyung Hee University, Seoul, South Korea

**Keywords:** synergistic effect, prostate cancer, natural product, scoping review, complementary and alternative medicine, combination therapy

## Abstract

**Background:** Prostate cancer is the second most common cancer in men and has the fourth highest mortality among men worldwide. Different combination therapies for cancer are being tested, and among them, the integration of natural products is increasing. This study reviews research on the combination of anticancer drugs and natural products for the treatment of prostate cancer and suggests future directions in this field.

**Methods:** Articles were identified by searching the PubMed, Embase, and Cochrane Library databases. Search keywords included the following: “Antineoplastic agents,” “Anticancer drug,” “Phytotherapy,” “Natural product,” “Drug synergism,” and “Synergistic effect”. The selection process focused on whether the differences in efficacy of anticancer drugs were evaluated when combined with natural products.

**Results:** Nineteen studies were included. All 19 studies evaluated efficacy *in vitro*, as well as 10 *in vivo*. There were 13 studies on a single compound extracted from natural products, three studies on mushroom and herb extracts, and three studies on herbal medicines consisting of three herbs, and a dietary supplement containing 10 herbs. Cancer cell lines used were PC-3 in nine studies, LNCaP in six studies, C4-2 in five studies, DU-145 in four studies, and 22Rv1 in two studies. Anti-cancer drugs co-administered were as follows: docetaxel in nine studies, doxorubicin and enzalutamide in three studies, paclitaxel and suberoylanilide hydroxamic acid in two studies, and cisplatin, vincristine, and bicalutamide in one study each.

**Conclusion:** Although prostate cancer is prevalent worldwide, there are relatively few studies on the use of natural products with anticancer agents as treatment. Since it has reported that the efficacy of anticancer drugs is enhanced by coadministration of natural products, it is necessary to conduct further studies on this.

## Introduction

Prostate cancer is the second most common cancer among men and has the fourth highest mortality among men worldwide (Ferlay J, 2020). The number of patients with prostate cancer is expected to increase from 1.41 million in 2020 to 2.43 million in 2040; therefore, prostate cancer poses a significant socioeconomic burden (Ferlay J, 2020). The 5-year relative survival rate of local or regional prostate cancer is as high as 99% or greater; however, the 5-year relative survival rate of distant prostate cancer is significantly lowered to 31%, which is of concern (American Cancer Society, 2022). Although the incidence and mortality rates of prostate cancer worldwide are decreasing or stabilizing (Culp et al., 2020), it is vital to develop a better treatment for prostate cancer because the incidence and mortality varies in each country, with prevalence increasing in some (Han et al., 2015).

Prostate cancer treatments include abiraterone and enzalutamide, which are anti-androgen medications, and docetaxel and cabazitaxel, which are cytotoxic agents that are sometimes used in combination with prednisone or radiotherapy (Cornford et al., 2017). For more effective treatment, various combination therapies are being tried for cancer treatment (Bayat Mokhtari et al., 2017); among them, the combination anticancer drugs and natural products are being studied (Lee et al., 2018; Choi et al., 2022). Natural products are not limited to the traditional herbal medicine in East Asia, including Korea and China, but are also commercialized or actively studied as botanical drugs in Europe and America. Since further development in the use of chemical is slow, interest in the use of natural products has been increasing (Takebe et al., 2018; Newman and Cragg, 2020). Natural products as cancer treatments, including for prostate cancer have also been researched and developed, but some challenges remain (Sanna et al., 2013; Huang et al., 2021; Singla et al., 2021a). Although many technological advances have been made, there are still natural products that are difficult to standardize and determine bioavailability (Atanasov et al., 2021), and despite many successful developments in the past, support for developing natural products from pharmaceutical companies and governments is decreasing (Newman and Cragg, 2020).

Approaches to the use of natural products may be broadly divided into three categories: extracting a single active ingredient or multiple active ingredients from a single natural product, a whole extraction from a single natural product, and a compound prescription composed of several natural products. Research on single ingredients is being conducted in many countries, especially in the United States and Europe, while research on compound prescriptions is mainly conducted in East Asia. This was influenced by the use of many traditional herbal medicines in Korea and China. Research methods, approval processes, and expected effects may differ depending on the category of the combined natural products. Therefore, it is necessary to investigate the current status of combination therapies for prostate cancer by classifying them according to the natural products they contain. Until now, clinical studies on the administration of natural products to patients with cancer have focused more on the management of accompanying symptoms or adverse effects of anticancer drugs on cancer patients, rather than addressing the cancer itself. To increase the use of natural products as anticancer drugs, clinical studies investigating their anticancer efficacy are required. Furthermore, to provide basic data for this, it is necessary to summarize the natural products used in coadministration studies conducted, in order to evaluate their anticancer efficacy. Many studies on the efficacy of bioactive natural compounds on prostate cancer have been published, and several review papers summarizing them have been published (Singla et al., 2021b; Miranda et al., 2022). However, so far, only reviews of natural products as single-agents have been conducted, and there have been no papers reviewing the synergistic effect of anticancer efficacy by coadministering natural products with anticancer agents. Therefore, in this study, studies that evaluated the synergistic effect of the combination of anticancer drugs and natural products were reviewed.

This study reviewed the studies on the combination of anticancer drugs and natural products for prostate cancer. We investigated what natural products, cancer cell lines, and anticancer drugs were studied. Therefore, this study aims to drive future research on treatment of prostate cancer with natural products, in combination with anticancer drugs.

## Materials and methods

### Study design

This study is a scoping review of studies that evaluated whether anticancer efficacy was increased with combination of anticancer drugs and natural products for treating prostate cancer. This study followed the PRISMA extension for scoping reviews (Tricco et al., 2018).

### Eligibility criteria

This study included only published literature; there was no restriction on the year of publication, and only literature published in English was included. Articles that evaluated the efficacy of the combination of natural products by presenting the anticancer efficacy of a single administration group of anticancer drugs and the combined administration group of natural products were selected.

### Information sources

Literature searches were conducted using PubMed, Embase, and Cochrane Library databases. The most recent search was conducted in July 2022.

### Search

The following free text keywords and Medical Subject Heading terms were used: “Antineoplastic Agents,” “Phytotherapy,” and “Drug Synergism.” Search histories are presented in Table 1. This example shows how to search the PubMed database; we used a similar search strategy for the other databases.

**TABLE 1:** Search history of PubMed.

Search number	Keywords
1	Antineoplastic agents [MeSH Terms]
2	Anticancer agent
3	Anticancer drug
4	Chemotherapy
5	Antitumor drug
6	Antitumor agent
7	Target therapy
8	Targeted therapy
9	Immunotherapy
10	#1 or #2 or #4 or #5 or #6 or #7 or #8 or #9
11	Phytotherapy (MeSH Terms)
12	Herbal medicine
13	Botanical drug
14	Natural product
15	Herbal therapy
16	Herb therapy
17	#10 or #11 or #12 or #13 or #15 or #16
18	Drug synergism (MeSH Terms)
19	Synergism
20	Drug augmentation
21	Drug Potentiation
22	Synergistic effect
23	Synergistic interaction
24	#18 or #19 or #20 or #21 or #22 or #23
25	#10 and #17 and #24

### Selection of sources of evidence

Duplicate literature was removed from search results across databases, and the titles and abstracts were checked first to select literature according to the selection/exclusion criteria. We then checked the full text of the selected literature and included studies that met the inclusion criteria in the final review. Two researchers independently performed this process, with disagreements resolved through discussion.

### Data charting process

To decide which variables to extract, two reviewers collaborated to create a data-charting form. We have created a data-charting form in an Excel file format. In an iterative procedure, the two reviewers independently plotted data, reviewed the findings, and revised the data charting form.

### Data items

The following information was extracted from each article: first author, year of publication, study type, cancer type, cell line type, intervention (natural product), intervention type, the raw material (if intervention is a single compound), combined anticancer agents, outcomes, and major findings. The study types were *in vivo*, *in vitro*, and a combination of both. The interventions used were single compound, extract, and compound prescriptions. The outcomes included only the evaluation of anticancer efficacy.

### Synthesis of results

A qualitative analysis was conducted because various studies on prostate cancer were included. A tabular format was used to summarize the data from all of the included studies. We grouped and summarized the included literature according to their study type, intervention type, and concomitant anticancer drugs.

## Results

### Selection of sources of evidence

**FIGURE 1:**
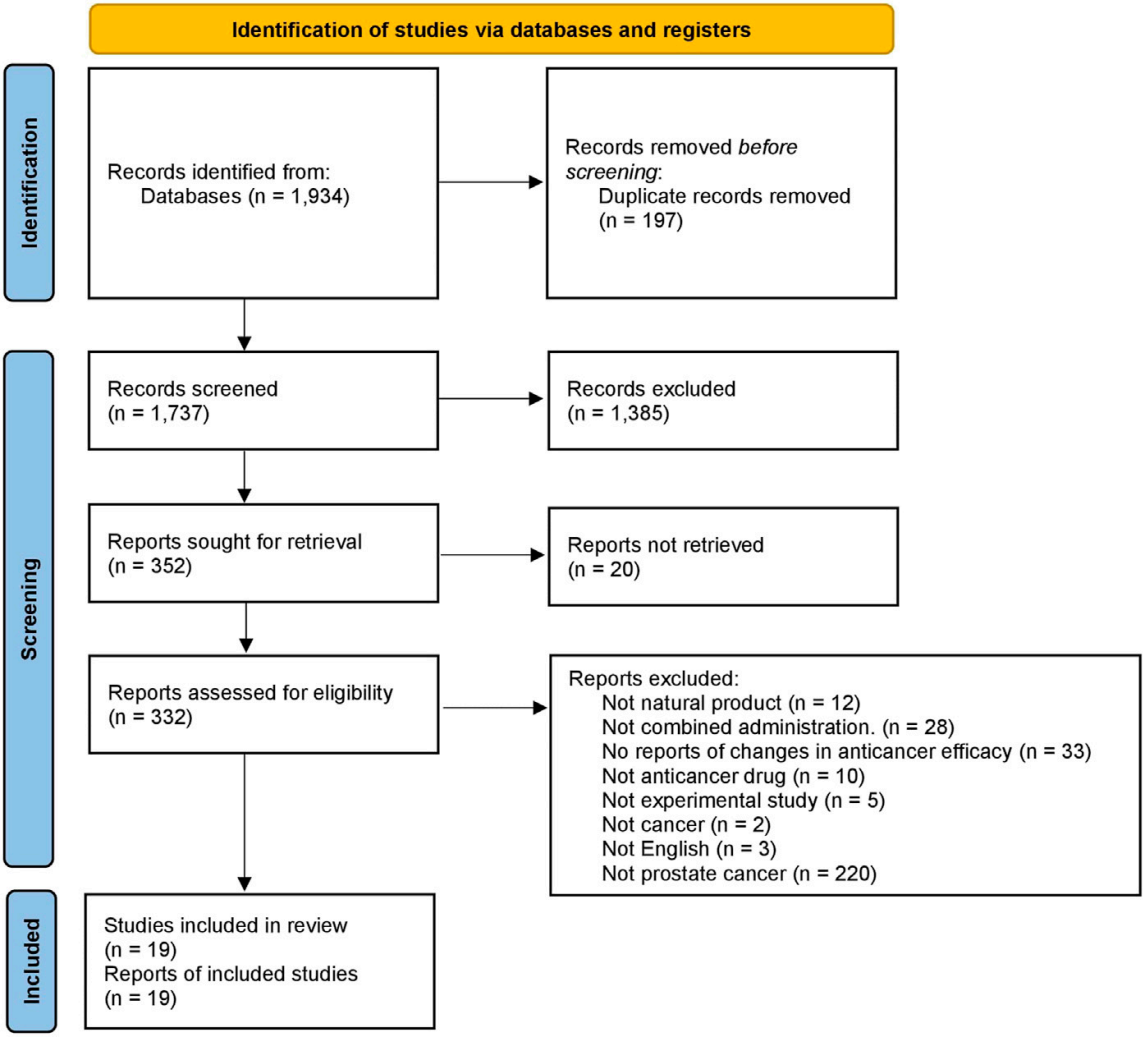
PRISMA flow diagram of study selection process.

After removing duplicates, 1,737 articles were extracted from both electronic biomedical databases and manual searches. Based on their titles and the abstract, 1,385 articles were excluded, with 352 articles to be retrieved. Of these, 19 proceedings and one study that we were unable to retrieve were excluded, and 332 full-text articles were assessed for eligibility. Of these, 313 were excluded for the following reasons: 12 did not investigate natural products, 28 did not administer natural and chemical therapy in combination, 33 did not report changes in anticancer efficacy, 10 did not use anticancer drugs, five were not experimental studies, two did not report cancer, three were not written in English, and 220 did not investigated prostate cancer. The remaining 19 studies were considered eligible for this review. The study selection process is illustrated in Figure 1. Based on the PRISMA flow diagram.

### Characteristics of sources of evidence

#### Single active ingredients

The intervention, study design, cell lines, combined anticancer drugs, and outcomes of studies on compounds extracted from natural products are presented in Table 2.

**TABLE 2:** Summary of studies using single active ingredient.

Intervention type	Intervention	Study design	Cell line	Combined anticancer drugs	Outcomes	References
Compound	Alpha-tomatine	*in vitro* & *in vivo*	PC3	paclitaxel	Cell viability, tumor volume	Lee et al. (2013)
Compound	Platycodin D	*in vitro*	DU145	docetaxel	Cell proliferation	Jin et al. (2021)
Compound	Retigeric acid B	*in vitro*	PC3, DU145	Cisplatin, doxorubicin, docetaxel, vincristine	Cell viability	Liu et al. (2018)
Compound	Quercetin	*in vitro* & *in vivo*	PC3	paclitaxel	Cell viability, tumor volume	Zhang et al. (2020)
Compound	Tricin	*in vitro*	PC3	docetaxel	Cell viability	Ghasemi et al. (2019)
Compound	Myricetin	*in vitro* & *in vivo*	C4-2B	enzalutamide	Cell viability, tumor volume	Liu et al. (2022)
Compound	Triptolide	*in vitro* & *in vivo*	C4-2R, 22Rv1	enzalutamide	Cell viability, tumor volume, tumor weight	Han et al. (2017)
Compound	Sulforaphane	*in vitro*	22Rv1	enzalutamide	Cell viability	Khurana et al. (2017)
Compound	Resveratrol	*in vitro*	DU145, LNCaP	SAHA	Apoptosis	Kai et al. (2010)
Compound	Pterostilbene	*in vitro* & *in vivo*	LNCaP, PC3	SAHA	Cell viability, tumor growth	Butt et al. (2017)
Compound	Capsaicin	*in vitro* & *in vivo*	LNCaP, PC3	docetaxel	Cell viability, tumor volume, tumor weight	Sánchez et al. (2019)
Compound	Ginsenoside	*in vitro* & *in vivo*	LNCaP, PC3	docetaxel, gemcitabine	Tumor weight	Wang et al. (2008)
Compound	Honokiol	*in vitro* & *in vivo*	C4-2	docetaxel	Cell viability, tumor areas	Shigemura et al. (2007)

SAHA, suberoylanilide hydroxamic acid.

Alpha-tomatine, a major saponin found in tomatoes, has been known to exhibit anti-tumor activity in human prostate cancer cells. Lee et al. (2013) showed that when paclitaxel and alpha-tomatine were administered in combination, cell viability in PC-3 cells, and tumor volume in BALB/c mice inoculated with PC-3 cells were significantly reduced. Furthermore, combination treatment had no cytotoxic effect on RWPE-1 cells, which are normal human prostate cells. It has been reported that the anticancer efficacy of this combination therapy is related to inhibition of PI3K/Akt signaling.

Platycodin D, a major bioactive compound derived from Platycodonis radix, has been widely used in traditional East Asian medicine to relieve respiratory discomfort. Recently, several studies have focused on the anticancer efficacy of platycodin D. Jin et al. (2021) reported that platycodin D combined with docetaxel synergistically suppressed the growth of DU-145, prostate cancer cells. This action by the regulation of autophagy and apoptosis appears to be mainly due to EGFR-mediated pathways; reactive oxygen species (ROS), AMP-activated protein kinase, and P38 pathways are also presumed to have contributed.

Retigeric acid B, a natural pentacyclic triterpene acid isolated from the Lobaria, is a promising anticancer agent in prostate cancer cells. Liu et al. (2018) have reported that when retigeric acid B was combined with cisplatin, which is also an anticancer treatment, including against prostate cancer, anticancer efficacy increased against PC-3 and DU-145, prostate cancer cells. Doxorubicin, etoposide, docetaxel, and vincristine were tested in combination. No significant increase in cytotoxicity was observed in the DU-145 cells. In PC-3 cells, a significant synergistic effect was observed in combination with doxorubicin; however, the increase in anticancer efficacy was greater when combined with cisplatin. This action appears to be related to the inhibition of DNA repair and activation of the death receptor 5.

Quercetin, a flavonoid found in many plants, such as fruits, vegetables, and grains, has been shown to inhibit the growth of various types of cancers. Zhang et al. (2020) have investigated whether quercetin increased the efficacy of paclitaxel in PC-3 prostate cancer cells and reported that it inhibited cell proliferation, increased apoptosis, arrested the cell cycle, and inhibited cell migration. In PC-3 cancer-bearing mice, tumor volume growth was effectively inhibited in the groups administered with quercetin and paclitaxel; moreover, there was no significant difference in body weight changes. It has been reported that ROS production, estrogen receptor stress induction, and hnRNPA1 downregulation are involved in anticancer efficacy.

Tricin is a flavonoid isolated from *Allium atroviolaceum* and is known to have antioxidant, anti-influenza, and hepatoprotective effects. Ghasemi et al. (2019) investigated whether tricin potentiated the anticancer effect of docetaxel in PC-3 cells. Co-treatment with tricin and docetaxel inhibited cell proliferation, even at sub-effective doses. Overexpression of MiR-21, which is associated with metastasis and drug resistance in prostate cancer, was decreased in tricin-treated cells.

Myricetin is a flavonoid compound, with anti-oxidative, anti-inflammatory, and anti-cancer properties. Liu et al. (2022) have investigated whether myricetin plus enzalutamide inhibited tumor volume growth. When myricetin was used in combination with Enzalutamide, an inhibitor of the androgen receptor, it was reported to increase cytotoxicity and decrease tumor growth significantly. In this study, to increase the bioavailability of myricetin, it was encapsulated by poly lactic-co-glycolic acid (PLGA), a drug carrier.

Triptolide is one of the main active compounds extracted from the medicinal herb *Tripterygium wilfordii*. Han et al. (2017) have reported that when triptolide was combined with enzalutamide synergistically suppresses cell growth and inhibits tumor progression. It has been reported that higher levels of the cleaved products of Caspase-3 and PARP and disrupting the phosphorylation of AR are involved in anticancer efficacy.

Sulforaphane, a phytochemical present in cruciferous vegetables, is a promising anticancer agent in various cancer cells. Khurana et al. (2017) showed that when sulforaphane and enzalutamide were administered in combination, cell viability in 22Rv1 cells was significantly reduced. Decreases in full-length AR and AR-V7 levels and increases in ubiquitination and proteasomal activity have been reported to be related to this effect.

Resveratrol is a polyphenol contained in grapes, peanuts, and berries, and is known to have various effects such as cardioprotective, anti-inflammatory, chemopreventive, and anticancer. Kai et al. (2010) have reported that resveratrol combined with suberoylanilide hydroxamic acid (SAHA) synergistically increased apoptosis in DU145 and LNCaP cells. Metastasis-associated protein 1 downregulation leading to increased p53 acetylation and activating the pro-apoptotic gene has been reported as a potential mechanism of this effect.

Pterostilbene, a natural stilbene found in grapes and blueberries, has been reported to have chemo-protective and anti-inflammatory capabilities (Koh et al., 2022). Butt et al. (2017) have reported that when pterostilbene was combined with SAHA, cell viability, and tumor growth were inhibited against LNCaP and PC-3 cells. This action appears to be related to the reduction of the metastasis-associated protein 1-associated proangiogenic factors HIF-1α, VEGF, and IL-1β causing a reduction in angiogenesis.

Capsaicin, famous for the spicy ingredient of hot chili peppers, has been reported to have anticancer effects on several cancer cells. Sánchez et al. (2019) have investigated whether capsaicin increased the efficacy of docetaxel in LNCaP and PC3 cells and reported that it inhibited cell viability and tumor growth. It has been reported that higher AMP-activated kinase activity and decreased Akt phosphorylation are involved in the synergistic effect.

Ginsenoside is a component of ginseng, a widely used herb, and many efficacy studies have been conducted. Wang et al. (2008) investigated whether ginsenoside potentiated the anticancer efficacy of docetaxel and gemcitabine in LNCaP and PC3 cells. Ginsenoside significantly increased tumor growth inhibition when combined with docetaxel or gemcitabine. When combined with radiation therapy, the anticancer effect has not been significantly increased.

Honokiol, a lignan isolated from magnolia tree, is known to have anticancer efficacy. Shigemura et al. (2007) have reported that when honokiol was combined with docetaxel synergistically inhibits cell viability and decreases tumor areas in C4-2 xenograft model. It has been reported that the combination of anticancer drugs and honokiol can be expected to overcome multiple drug resistance and have antiangiogenic effects.

#### Whole extraction from a single natural product

Three studies have reported that the whole extract of a natural product could be used in combination with anticancer drugs for prostate cancer (Table 3).

**TABLE 3:** Summary of studies using whole extraction from a single natural product.

Intervention type	Intervention	Study design	Cell line	Combined anticancer drugs	Outcomes	References
Extract	*Phellinus linteus*	*in vitro*	LNCaP	doxorubicin	apoptosis	Collins et al. (2006)
Extract	*Wedelia chinensis*	*in vitro* & *in vivo*	PC3, DU145	docetaxel	Cell proliferation, tumor volume	Tsai et al. (2017)
Extract	*Beta vulgaris*	*in vitro*	PC3	doxorubicin	Cell viability	Kapadia et al. (2013)

*Phellinus linteus* is a mushroom mainly composed of polysaccharides with antitumor activity. Collins et al. investigated the effect of powdered *P. linteus* on apoptosis in prostate cancer LNCaP cells (Collins et al., 2006). *P. linteus* and doxorubicin were found to induce apoptosis when administered in combination at doses that did not induce apoptosis in monotherapy. *P. linteus* is presumed to induce apoptosis through JNK activation, a c-Jun N-terminal kinase.

*Wedelia chinensis* is an herb reported to have anticancer efficacy against prostate cancer. It has been reported that the mechanism is related to the inhibition of androgen receptor signaling. Tsai et al. (2017) investigated whether the co-administration of *W. chinensis* and docetaxel increases anticancer efficacy. As a result of evaluating the effects on docetaxel efficacy of luteolin, apigenin, and wedelolactone, the components of *W. chinensis,* only wedelolactone significantly increased cytotoxicity. In addition, *W. chinensis* has been shown to reduce docetaxel-mediated tissue damage.

*Beta vulgaris* is a plant eaten in many countries and is commonly known as a beet. Kapadia et al. (2013) conducted a study in which *B. vulgaris* was co-treated with doxorubicin on pancreatic, breast, and prostate cancer cells. When *B. vulgaris* was combined with doxorubicin, the cytotoxicity to pancreatic and breast cancer cells showed synergism, but not in prostate cancer cells. It has been reported that the combined use of *B. vulgaris* enables the dosage reduction of anticancer drugs, thereby alleviating adverse effects.

#### Compound prescription

In three studies, two mixtures of a natural product extracts were used in combination with anticancer drugs for prostate cancer (Table 4).

**TABLE 4:** Summary of studies using compound prescription.

Intervention type	Intervention	Study design	Cell line	Combined anticancer drugs	Outcomes	References
Formula	Aneustat (OMN54)	*in vitro* & *in vivo*	C4-2	docetaxel	Apoptosis, tumor volume	Qu et al. (2014)
Formula	Aneustat (OMN54)	*in vitro* & *in vivo*	C4-2	docetaxel	Cell proliferation, cell migration	Qu et al. (2018)
Formula	Zyflamend	*in vitro*	LNCaP	bicalutamide	Cell viability, cell proliferation	Yan et al. (2012)

Aneustat (OMN54) is an herbal formulation containing *Ganoderma lucidum*, *Salvia miltiorrhiza*, and *Scutellaria barbata*. It has the potential to have immunomodulatory, antiangiogenic, anti-inflammatory, antiproliferative, and antiviral properties. Qu et al. (2014) conducted a study in which Aneustat and docetaxel were administered in combination to a patient-derived advanced prostate cancer tissue xenograft model. Coadministration enhanced anti-tumor activity remarkedly without inducing major toxicity. Inhibition of both androgen receptor expression and AKT phosphorylation in coadministration appears to be involved in this activity.

The same researchers further conducted a study on the coadministration of Aneustat with docetaxel in prostate cancer (Qu et al., 2018). The combination of Docetaxel and Aneustat was confirmed to have a synergistic effect on inhibiting prostate cancer cell proliferation. It was also reported that it inhibited the metastasis of prostate cancer cells to the lungs. Downregulation of the *FoxM1* gene, a regulator of tumor metastasis, was reported as a potential mechanism of anti-metastatic effect.

Zyflamend is a combination of herbal extracts containing ginger, rosemary, turmeric, Chinese goldthread, holy basil, Hu Zhang, barberry, oregano, green tea, skullcap (Huang et al., 2014). Zyflamend has been reported to have anti-cancer and anti-inflammatory effects, and many efficacy studies have been conducted on prostate cancer (Lin et al., 2019). Yan et al. (2012) have investigated whether Zyflamend increased the efficacy of bicalutamide in LNCaP cells and reported that it reduced cell growth, induced apoptosis. Inhibition of Bcl-2 and Bcl-xL, induction of cleavage of Caspase-3 and PARP protein, repression of AR expression level and Nkx3.1 protein level, and reduction of PSA secretion appears to be involved in this co-treatment.

## Discussion

A scoping review was conducted on studies that evaluated whether the anticancer efficacy increased through the combined treatment with natural products and anticancer drugs for prostate cancer. All 19 studies included were conducted *in vitro*, while 10 of them were also conducted *in vivo*. As efficacy and safety studies in animal models are required before clinical trials are conducted, *in vivo* studies should be conducted later.

Most of the studies are on single compounds, and the reason why there are few studies on compound prescriptions seems to be that compound prescriptions of natural products are mainly interested in some parts of East Asia, such as Korea and China (Kim and Kang, 2020; Seo et al., 2021). These days, multicomponent drugs that hit multiple targets are attracting attention. Moreover, whole extract from a single natural product and compound prescription contain various ingredients, thus multi-target cancer treatment is possible (Chang et al., 2020; Lu et al., 2022). Therefore, it is necessary to pay more attention to cancer treatment using natural products.

The most commonly used cell line was PC-3, followed by LNCaP in six cases, C4-2 in five cases, DU-145 in four cases, and in two case in 22Rv1. PC-3 and DU-145 are representative cell lines of prostate cancer in which androgen receptors are not expressed. PC-3 has a normal androgen receptor gene and DU-145 has a methylated androgen receptor gene (Chlenski et al., 2001). The LNCaP cell line, which is widely used for comparison with PC-3, is derived from lymph nodes and is characterized by the expression of androgen receptors and prostate-specific antigens (van Bokhoven et al., 2003). C4-2 is a cell line derived from LNCaP, which exhibits androgen-independent growth associated with bone metastasis (Liu et al., 2004). The C4-2R cells used in the triptolide study were made resistant to enzalutamide by chronically exposing C4-2 cells to enzalutamide (Han et al., 2017). 22Rv1 cell line was derived from a xenograft, representing an AR-positive prostate cancer model, demonstrating AR-dependent and AR-independent growth (Sramkoski et al., 1999). Experiments have been conducted on various cell lines; however, few experiments have been performed on multiple cell lines for one natural product.

The combined anticancer drugs were docetaxel in nine cases, doxorubicin and enzalutamide in three cases, paclitaxel and SAHA in two cases, and cisplatin, vincristine and bicalutamide in one case. Since docetaxel has long been used as a major anticancer agent for the treatment of prostate cancer and has been suggested as a standard treatment at an early stage, it has been widely used in combination studies (Puente et al., 2017). There were fewer studies using androgen receptor targeted agents which are commonly used for prostate cancer treatment, in combination with natural products compared to chemotherapy (Gillessen et al., 2022). This is considered to be because chemotherapy used for various cancers was first selected as a target for combination. This suggests that there is a lot of need for studies on natural products combined with specific treatments for each cancer in the future.

Studies on the combined administration of natural products and anticancer drugs for prostate cancer are limited compared to those for other cancer types, and in most cases, additional combined administration was conducted while researching on the anticancer efficacy of natural products. For the coadministration of natural products to prostate cancer patients in clinical practice, studies should be conducted to comprehensively evaluate the efficacy of combining natural products with various drugs in various cell lines. The development of new drugs using natural products had temporarily slowed down due to technical limitations, but these have been overcome, and is gaining attention once again (Atanasov et al., 2021). This study may serve as a basis for future studies regarding the combination of drugs with natural products in managing patients with prostate cancer.

This review shows that new approaches to coadministration studies are being tried. The study of myricetin selected histone lysine demethylase family 4 inhibitor through structure-based screening, and the use of PLGA-encapsulated myricetin to increase bioavailability is also unique (Liu et al., 2022). Although marker compounds of many natural products have been identified, this does not mean that they are biologically active compounds, and there are some compounds with low bioavailability (Ministry of Food and Drug Safety, 2021). Compounds with low bioavailability can be considered in combination with carriers that increase bioavailability (Parveen et al., 2022; Zou et al., 2022). There was a study conducted on resistant cancer cell lines, which shows the possibility of natural products to overcome the drug resistance in cancer (Han et al., 2017). This suggests that studies on the combined administration of natural products to overcome anticancer drug resistance can also be an important way for anticancer treatment using natural products.

There are two main reasons for the small number of articles included in our review among 1,700 articles searched. The first is the search method. Although we are interested in prostate cancer, there are quite a few studies that are not included in the combination studies of our interest when “prostate cancer” is included in the search keyword. Therefore, we conducted a literature search using coadministration and cancer-related keywords and selected prostate cancer research in the selection process. As a result, only a small number of articles were included compared to the number of screening articles. The second is that there are fewer studies on new drugs for prostate cancer compared to other cancers. Prostate cancer has a higher 5-year survival rate than other cancers. As a result, the priority of developing new drugs is falling behind compared to other cancers with high mortality. However, the 5-year survival rate of metastatic prostate cancer is drastically reduced to 30%, and the risk factor for prostate cancer, older men, is increasing significantly with the increase in lifespan, so it is very important to research treatment for prostate cancer (American Cancer Society, 2022).

Natural products are attracting attention as new therapeutic agents for various types of cancer, and many studies have been published on prostate cancer. However, interest in combination administration, which is covered in this study, is even more important when considering the reality of new drug development. In order to be approved as a new drug, a confirmatory clinical trial must be conducted, and to do so, many patients with cancer must participate in the clinical trial. Although the system may vary slightly from country to country, it is generally an ethical issue to test a medicine that has not been fully validated when alternative treatments are available for patients with severe diseases (Temple and Ellenberg, 2000). Also, physicians participating in the study are reluctant to administer the test drug to investigate the effectiveness of the test drug when there are alternative treatments. Therefore, it is very difficult to conduct a clinical trial to administer a new drug, especially a natural product alone, or it is difficult to confirm the effectiveness because it is only for terminal patients with cancer who do not respond to existing treatments. This situation eventually lowers the success rate of developing anticancer drugs as a single treatment for natural products. Considering these realistic conditions, research on natural products as a combination therapy is important because it can be an avenue for new drug development.

This study has several limitations. First, most anticancer treatments in combination with natural products were chemotherapies. Few studies have been conducted in combination with androgen deprivation therapy or targeted therapy, which is widely used in the treatment of prostate cancer recently (Gillessen et al., 2022). This may also be partially due to the limited search keywords, but it is presumably because chemotherapy such as docetaxel was the most widely used treatment for prostate cancer as there are not many combination studies on other treatments. In the future, research on the combination of natural products for more diverse treatments is required. Second, because of the wide variety of interventions, individual drug or natural product names were not included in the search keywords. Although a comprehensive search has been conducted as much as possible, there may be some missing studies using natural products and anticancer drugs in combination. Despite these limitations, this study will contribute to suggesting a natural product combination therapy for prostate cancer in the future, as it summarizes studies showing synergistic effects of the combination of natural products and anticancer agents, which are mostly considered for the treatment of prostate cancer. Based on our findings, it is expected that *in vivo* studies and clinical trials that administer natural products together with drugs against prostate cancer may be conducted.

## Data Availability

The original contributions presented in the study are included in the article/supplementary material, further inquiries can be directed to the corresponding author.
